# Similarities and differences between nigral and enteric dopaminergic neurons unravel distinctive involvement in Parkinson’s disease

**DOI:** 10.1038/s41531-022-00308-9

**Published:** 2022-04-22

**Authors:** Alcmène Chalazonitis, Meenakshi Rao, David Sulzer

**Affiliations:** 1 Department of Pathology & Cell Biology, Vagelos College of Physicians & Surgeons, Columbia University, New York, NY USA; 2 Department of Pediatrics, Boston Children’s Hospital and Harvard Medical School, Boston, MA USA; 3 Departments of Psychiatry, Neurology, and Pharmacology, Division of Molecular Therapeutics, New York State Psychiatry Institute, Columbia University, New York, NY USA; 4 Aligning Science Across Parkinson’s (ASAP) Collaborative Research Network, Chevy Chase, MD 20815 USA

**Keywords:** Parkinson's disease, Constipation

## Abstract

In addition to the well-known degeneration of midbrain dopaminergic neurons, enteric neurons can also be affected in neurodegenerative disorders such as Parkinson’s disease (PD). Dopaminergic neurons have recently been identified in the enteric nervous system (ENS). While ENS dopaminergic neurons have been shown to degenerate in genetic mouse models of PD, analyses of their survival in enteric biopsies of PD patients have provided inconsistent results to date. In this context, this review seeks to highlight the distinctive and shared factors and properties that control the evolution of these two sets of dopaminergic neurons from neuronal precursors to aging neurons. Although their cellular sources and developmental times of origin differ, midbrain and ENS dopaminergic neurons express many transcription factors in common and their respective environments express similar neurotrophic molecules. For example, Foxa2 and Sox6 are expressed by both populations to promote the specification, differentiation, and long-term maintenance of the dopaminergic phenotype. Both populations exhibit sustained patterns of excitability that drive intrinsic vulnerability over time. In disorders such as PD, colon biopsies have revealed aggregation of alpha-synuclein in the submucosal plexus where dopaminergic neurons reside and lack blood barrier protection. Thus, these enteric neurons may be more susceptible to neurotoxic insults and aggregation of α-synuclein that spreads from gut to midbrain. Under sustained stress, inefficient autophagy leads to neurodegeneration, GI motility dysfunction, and PD symptoms. Recent findings suggest that novel neurotrophic factors such as CDNF have the potential to be used as neuroprotective agents to prevent and treat ENS symptoms of PD.

## Introduction

The enteric nervous system (ENS) is the only subdivision of the peripheral nervous system (PNS) that encompasses neuronal phenotypes as diverse as those in the central nervous system (CNS). For example, neurotransmitters expressed in CNS and ENS neurons but not in extra-enteric PNS ganglia include serotonin (5-HT)^1^, gamma-aminobutyric acid (GABA)^2,3^, and dopamine (DA)^4^. Advances in characterizing the signals underlying the specification and development of midbrain dopaminergic neurons have been recently described in several comprehensive reviews^5–7^. Similar characterization of neuronal diversification in the ENS, particularly with respect to the dopaminergic phenotype, has lagged^8–10^. Recent single-cell sequencing analyses, however, have now identified at least 12 neuronal subclasses in the myenteric plexus (MyP) of the intestine, including a tyrosine hydroxylase (TH)-expressing population^3,11,12^. Recent evidence indicates that specific enteric, as well as midbrain and other CNS neurons, can be affected in neurodegenerative diseases including Parkinson’s disease (PD)^13^. Furthermore, newly identified factors expressed in both midbrain and enteric neurons have been shown to be neuroprotective in mouse models of PD.

A motivation for this comparative review is to assess whether, independent of their location or function, it is sufficient for neurons to be dopaminergic to be selectively vulnerable during the progression of PD. We review similarities and differences between the dopaminergic neurons in the ventral midbrain substantia nigra pars compacta (SNpc) and ventral tegmental area (VTA) with those of the ENS. We discuss how these neuronal populations evolve from early development to maturity and older age in light of mechanisms known to promote their specification, differentiation, maintenance, function, and vulnerability. While PD is primarily a disease of the aged, early developmental traits are considered here because identification of susceptibility genes expressed prior to maturity could reveal subsequent disease mechanisms. A better understanding of mechanisms shared by both populations may assist in the design of therapies that would protect and restore the function of both the ENS and the midbrain in PD.

## Dopaminergic neurons develop later in the ENS than in the SNpc

### Midbrain—regional patterning, specification, and cell fate commitment (E7.5–E10.5)

Information on the unraveling of the molecular interactions controlling these developmental stages is described in detail in a recent review^7^. All midbrain dopaminergic neurons originate from the mesodiencephalic floor plate (FP). At embryonic day 7.5 (E7.5; developmental ages cited are for mice except where specified) the isthmus/midbrain-hindbrain border develops under the reciprocal balanced expression of Otx2 (in the midbrain) and GbX2 (in hindbrain). Within the isthmus, Fgf8 is expressed as is Wnt-1, the latter being also expressed within the midbrain plays a major role in dopaminergic neurons development. Moreover, the transcription factor (TF) Engrailed 1 (En-1) drives the consolidation of the isthmus by controlling the proper expression of Fgf8, Wnt-1, and Otx2. This cross-activation of Otx2 with Wnt-1 is necessary to activate the genetic networks for the dopaminergic commitment and to inhibit alternative phenotypes such as the serotonergic^14,15^.

At E8, the Gli2A-mediated secreted factor Sonic Hedgehog (SHH) specifies the FP via induction of the TF Foxa2/HNF-3 beta^6,16^ At E8.5, SHH allows correct patterning of the FP, and the dopaminergic neurons progenitors^17,18^, while Wnt-1 allows an expansion of the pool of neurons progenitors in the VZ of the developing midbrain by inhibiting SHH. At E9.5, the neuronal progenitors become committed to the dopaminergic phenotype and start expressing the TFs Lmx1a, 1b, Foxa1, Foxa2, Msx1/2, Ascl1/Mash1, and Ngn2^19–21^. The glycoprotein DKK3, a modulator of Wnt-1 and beta-catenin signaling, activates Lmx1a and Pitx3 which promotes the differentiation of the rostrolateral subset of dopaminergic neurons in the SNpc^22^.

At E10.5, the dopaminergic neurons start being born and the complex cascade of TFs interacting in a time-dependent manner leads to specific dopaminergic neuron subtypes. The A8, A9, and A10 subgroups of progenitors differentiate respectively into the retrorubral field (RrF), the SNpc, and the VTA^23^. Sox6 specifies and localizes to SNpC neurons while Otx2 specifies and localizes to VTA neurons^24^. While both Ngn2 and Ascl1 are expressed in the ventral midbrain, only Ngn2 is required^21^. Foxa1 and Foxa2 cooperate with Lmx1a and Lmx1b to promote mesodiencephalic dopaminergic neuron development^25^. Both are required to regulate the progression of midbrain dopaminergic neuron maturation^26,27^. Foxa2 is not only expressed in precursors of mesodiencephalic dopaminergic neurons but also controls the birth and spontaneous degeneration of dopaminergic neurons during aging^28^. Foxa1 and Foxa2 reciprocally regulate SHH signaling in the specification of ventral midbrain progenitors^16^ and maintain the dopaminergic phenotype at late embryonic stages^29^. In addition, the orphan nuclear receptor Nurr1/Nr4a2 acts synergistically with Ngn2 to induce midbrain dopaminergic neurons from neural stem cells and control the expression of TH, the rate-limiting enzyme involved in dopamine production^30^.

### Midbrain—terminal differentiation and phenotypic maturation (E11–E18.5)

En-1 and Pitx3 are two TFs that reciprocally regulate the expression of mesodiencephalic genes in precursors of SNpc dopaminergic neurons^31,32^. By E11 in mice, 49% of the dopaminergic neuronal precursors in the SNpc have become post-mitotic^33^ and in rats by E12, about 80% of nigral dopamine neurons have been born^34^. From E12 to E14 in mice, Ebf1 is essential for the terminal migration of immature dopaminergic neurons of the ventral mesencephalon in the SNpc^20^. At E18.5, a number of additional genes (*Aldh1a1, Sox6, Calb1*^*Low*^) were identified in D2 receptor expressing embryonic neurons, to encode proteins involved in the maturation of midbrain dopaminergic neurons. Expression of these additional identified proteins has been validated by single-cell RNA sequencing (scRNA-seq) and immunocytochemistry, shown to be selective for TH-positive neurons, and found to persist postnatally in SNpc neurons^35^. More recently, scRNA-seq was performed on Lmx1a-expressing precursors of mesencephalic dopaminergic neurons as they were differentiating. High levels of expression of Lmx1a, Lmx1b, Corin, Wnt-1, TH, and Pitx3 were present between E10 and E13.5^36^. A similar approach was performed on Pitx3-expressing developing midbrain neurons between E13.5 and P90. In the ventral midbrain over this time period, seven subgroups were identified as expressing the DA uptake transporter (DAT)/Slc6a3, Th, Pitx3, Nxph4, Gad2, Aldh1a1, or Vip. This diversity was conserved in human ventral midbrain neurons. Both high and low DAT–expressing sub-lineages expressed Lmx1a, Lmx1b, Foxa2, and Nr4a2/Nurr1. A recent review comparing various scRNA-seq analyses points out that while all the dopaminergic lineages express DAT, identified subtypes can be found in different locations of the SNpc. For instance, the Aldh1a1 and Sox6 expressing dopaminergic neurons subtype is found in the ventral SNpc, while the subtype expressing Sox6 but not Aldh1a1 is located in the dorsal SNpc^37^.

### ENS—regional specification and cell fate commitment (E10–E14)

Enteric dopaminergic neurons become specified many days later than their midbrain counterparts. This delay occurs because their precursors are derived from the neural crest and are known as enteric neural crest-derived cells (ENCDCs). ENCDCs expressing PHOX2B, RET, and SOX10 originate from vagal, truncal, and sacral somatic levels. They must first enter the gut and then migrate long distances in a rostro-caudal or caudal-rostral fashion to colonize the entire length of the organ^38,39^. As they become post-mitotic, these precursors differentiate into neurons and glia and form the ganglia of the MyP, located in between the longitudinal and circular muscle layers. Then, in a secondary wave of migration, the ENCDC form the ganglia of the submucosal plexus (SMP) located in between the circular muscle and mucosa, closer to the gut lumen^40–42^.

The process of migration and sustained proliferation of the pool of ENCDC is controlled by several TFs and inductive molecules. The transcriptional regulator SOX10 maintains the pluripotency of ENCDC and inhibits their neuronal differentiation^43^. Another TF ZEB2 synergizes with SOX10 to regulate the balance between proliferation and differentiation of enteric precursor cells via Endothelin-3-EdnrB signaling^44^. A spontaneous dominant mutation in *Sox10* associated with megacolon (*Dom* mouse) was identified early on as a model of Hirschsprung’s disease, a congenital disorder in which children are born lacking enteric ganglia, typically in the distal colon. Both Sox10 and EdnrB expression become deregulated in heterozygous *Dom* mice leading to an absence of enteric ganglia in the distal colon^45^. Glial cell line-derived neurotrophic factor (GDNF) and its signaling co-receptor, the tyrosine kinase RET, are also essential for proper colonization and maintenance of the pool of migrating precursors along the fetal gut^46,47^. Many cases of both familial and sporadic Hirschsprung’s disease are associated with loss of function mutations in RET, while GDNF mutations are more rare^48^. Inactivating mutations in PHOX2B, a TF essential for the development of the ENS, also cause congenital aganglionosis in tandem with other defects in autonomic function. PHOX2B controls caudal migration of ENCDC in the mouse foregut, maintains expression of the TF ASCL1 (Mash1), and induces expression of both Ret and TH^49^.

In contrast with the midbrain, ASCL1, but not NGN2, is essential for neurogenesis and development of precursors of specific enteric neuronal subtypes, including TH+ neurons in at least some regions of the GI tract. In mice lacking ASCL1, the proportion of TH+ neurons in the MyP of the stomach is ~60% lower at E18.5 than that in wildtype controls^50^. One consideration that complicates the understanding of enteric dopaminergic neuron development is that there is a population of neuronal precursors in the developing ENS between E10 and E13 that is transiently catecholaminergic (TC), initially expressing TH and then downregulating it upon terminal differentiation^51^. These TC cells are ASCL1-dependent^52^. Because Ascl1 null mice were studied at E18.5, it is unlikely that their deficit in TH+ cells at this time point was due to simply having fewer TC cells. It remains unclear, however, to what extent transiently TH+ cells contribute to the final population of dopaminergic neurons. The evidence to date hints that any contribution is likely small, at least in the MyP of the small intestine. Single-cell profiling of the developing ENS indicates that most TH-expressing myenteric neurons in the small intestine are generated after birth^3^. Genetic fate-mapping of TH-expressing cells in mice designed to have both TH-Cre and Cre-dependent YFP reporter transgenes has shown that TH+ cells and their derivatives constitute less than 3% of neurons in the MyP of the ileum in adult mice, and only 5% of these YFP+ cells actively express TH^53^. These observations indicate that the majority of cells derived from TH-expressing TC progenitors become non-dopaminergic neurons^53^. Lineage tracing with an inducible reporter system that enables labeling TH-expressing cells and their descendants from embryonic and postnatal time points in the stomach, intestine, and colon would help to clarify the developmental origin of TH+ neurons in the adult ENS. Such a study would also elucidate to what degree gastric and intestinal TH+ neurons are comparable in terms of developmental trajectories.

Shh secreted from primitive gut endoderm is another important signal involved in ENS development that controls the proliferation and migration of the ENCDC. The TFs Gli1, Gli2, and Gli3 mediate Shh signaling output. Gli1 is a direct target gene of Shh and is upregulated by Shh. Because Gli2 has not been detected in ENCDC its regulation by Shh has not been analyzed in this context. In contrast, both activated and repressor forms of Gli3 are highly expressed in ENCDC and the primary activity of Shh signaling to ENCDC is to inhibit the repressor form of Gli3^54^. Three mutations in GLI have been identified in some Hirschsprung’s patients, with ensuing aberrant Shh levels due to a disruption of Sufu within the Sufu-Gli-Sox10 regulatory loop^55^. Shh also regulates the expression of the glial cell line-derived neurotrophic factor (GDNF) in the mesenchyme. In chick and mouse, hindgut epithelial-derived Shh interferes with ENCDC migration by increasing the expression of ECM molecules such as chondroitin sulfate proteoglycans and by decreasing GDNF. This in turn inhibits proliferation and induces differentiation of neuronal precursors^56^.

### ENS—terminal differentiation and phenotypic maturation (E15.5–P3)

Enteric dopaminergic neurons become specified and differentiate only after colonization of the gut is completed. A recent study using scRNA-seq to analyze the cellular composition of the small intestinal MyP at E15.5 and E18.5 suggested that enteric neuronal diversification occurs along two distinct trajectories to give rise to 12 transcriptionally distinct types of myenteric neurons^3^. Of these, only one type contained cells expressing TH (ENC11), and was presumed to arise mostly after birth. Interestingly, ENC11 neurons expressed *Th* and *Dbh* but not *Ddc*, implying that they were noradrenergic rather than dopaminergic. Transcriptional profiling studies of the adult mouse MyP have similarly failed to define a distinct dopaminergic population^11,12^. However, previous studies using qPCR and immunocytochemistry have shown clear evidence of DA, DAT, and DA receptor expression in postnatal and adult neurons of both enteric plexuses^4,57^. The disparity in these observations may reflect the limitations of scRNA-Seq to detect low abundance transcripts^58^ or the relative paucity of dopaminergic neurons in the MyP compared to the SmP, which has not been sampled in most of the scRNA-seq studies to date.

Since the TFs Foxa1 and Foxa2 are implicated in specification, development, maturation, and maintenance of midbrain dopaminergic neurons, could they also play similar roles in gut or ENS development? An early immunohistochemical study performed in embryonic and adult mouse tissue from many organs showed immunoreactivity of Foxa1 and Foxa2 localized in the gastric glands and in the epithelial crypts of Lieberkühn of small and large intestines. Ganglia of the ENS, however, were not examined^59^. It was recently reported that at E15–E16, ENCDC are a mix of precursors and post-mitotic TH neurons which already express Foxa2^57^. Most of the neurons in the small intestinal MyP that express TH in adult mice exit the cell cycle between E12.5 and P0 with a peak at E15.5^60^, while the majority in the SMP are born at E17.5 and continue to be born through P3^8^.

### Genes expressed in common by enteric and mesencephalic dopaminergic neurons

A comprehensive analysis of TFs and signaling molecules that regulate the fate and promote the development and diversification of enteric neurons permitted comparison of early (E11.5) and late (E15.5) gut fetal stages in the mouse^10^. Remarkably, by E18, enteric TH-labeled neurons express several genes in common with mesencephalic neurons (Ascl1, Ebf1, Sox6, Pbx3), and with neurons in the olfactory bulb and hypothalamus (Klf7, Satb1, Etv1), all of which are important for the development of the CNS dopaminergic phenotype. The conditional deletion of Sox6 resulted in a 70% reduction of the TH-expressing neurons in the stomach, accompanied by a reduced rate of gastric emptying, suggesting at least some key TFs are conserved. Foxd1 is one example of a TF shown to be expressed in most neurons of the stomach and intestines, including the TH-expressing neurons, but whose function has not been yet investigated^10^. The expression of the key TFs spanning fetal to postnatal stages E7.5–P90 in the mouse is compared in the SNpc and the ENS in Table 1. In addition to TFs, secreted signaling proteins (i.e., growth factors) also play a major role in the development of dopaminergic neurons in both the midbrain and the ENS environment.

**Table 1:** Compared transcription and secreted factors involved in patterning, specification, commitment, and differentiation of dopaminergic neurons in the midbrain and the enteric nervous system.

Midbrain-substantia nigra dopaminergic neurons				Enteric dopaminergic neurons			
Stage	TF/SF	Function	Refs	Stage	TF/SF	Function	Refs
E7.5– 10.5	Otx2 > GbX2	Patterning of isthmus	^7,14,15^	E9.5–13.5	Sox10	Maintain survival, pluripotency of ENCDC	^38,39,43^
	En-1 Fgf8, Wnt-1, Otx2	Consolidate progenitors domain and identity	^6,16,23^			Turn on expression of Phox2b and Ascl1	
	Shh--Foxa2	Specifies FP and patterning	^17–20^			Fetal gut colonization by ENCDC, proliferation, survival	^46,85^ Table 3
	Lmx1a,1b, Foxa1,a2, Msx1/2	Commitment to DAergic fate	^21,25–27^		GDNF	Control caudal migration of ENCDC in foregut	
	Ascl1/Mash1, Ngn2	Differentiation	^28,29^			Induce Ret and TH (transient) expression in stomach	
E11–12	Sox6, Otx2	Subpatterning and DAergic fate in RrF, SN, vs. VTA and rostrolateral differences	^24^		Phox2b	Present in all ENCDC as in TC precursors	^10,49^
	DKK3, Lmx1a, Pitx3,		^22^		Mash1/Ascl1	Required for specific phenotypes	^51,52^
	Nurr1, Ngn2	Synergize to induce TH expression	^30^		Ascl1 mutant	40% reduction of TH-neur. in gastric MyP	^50^
E12–14	En-1, Ptx3	Reciprocal regulation of expression of genes in precursors of SNpc	^31,32^	E10.5	Shh	Regulates GDNF	^56^
						Controls ENCDC proliferation, differentiation	
						Slows their migration	
		Become post-mitotic, 49% in mice, 80% in rat				Increases ECM	
						Decreases GDNF	
						Increases differentiation	
	Ebf1,	Terminal migration of immature DAergic neurons from the mesencephalon to the SNpc	^20^		Gli1,2,3	Regulated by Shh	^54^
						Gli -mutations in HSCPR deregulate Sufu-Gli-Sox10 loop	
						Aberrant Shh	^55^
E18.5	Aldh1a1, Sox6, Calb1^Low^	In D2 receptor expressing neurons	^24^	E10–13.5	Zeb2, Sox10	Synergize to regulate balance between proliferation and differentiation via Edn3-EdnrB	^44^
	Ebf1	Control maturation of Midbrain neurons	^35–37,73^	E15–16	Foxa2	Expressed by TH-labeled neurons in MyP	^10^
	Foxd1, Pbx3 Nr4a2, Bnc2, Pbx1	Persist postnatally in SNpc			Sox6	Expressed by TH neurons developing in vitro from ENCDC	^57^
P20–90	DAT-/Slc6a3	7 identified subgroups of DAT- neurons in dorsal SN	^37^	E15.5	Foxa2	Dopaminergic neurons birthdates: peak time, in MyP time range, in SMP	^60^
	Th, Pitx3, Nxph4, Gad2, Aldh1a1 or Vip		http://Mousebrain.org	E17.5–P3	Sox6,	Specify TH neurons in gastric MyP	^8^
				E18.5	Foxd1 Ebf1, Pbx3	Expressed in MyP, small intestine	^10^
					Satb1, Klf7, Etv1 Cux2, Zfp800, Klf7	Specification, differentiation, roles to be tested	^3^

## Similarity of signaling molecules that promote the development of dopaminergic neurons in the midbrain and the ENS

Many of the following signaling molecules as well as their receptors have been demonstrated to be necessary in the development of both midbrain and enteric dopaminergic neurons.

### The transforming growth factor-beta (TGF-beta)

TGF-beta which signals through TGF-beta R1 and TGF-beta R2, has been shown to cooperate with SHH and FGF8 in the induction, specification, differentiation, and survival of midbrain dopaminergic neurons^61,62^. The homeodomain interacting protein kinase 2 (HIPK2), a negative regulator of bone morphogenetic protein (BMP) signaling^63^, regulates the survival of midbrain dopaminergic neurons via the TGFbeta3–Smad-HIPK2 pathway as demonstrated in *Hipk2*^−/−^-mutant mice^64,65^. TGF-beta 1-3 and their receptors (TGF-beta R1-3) have been shown to be expressed in human and rat gut smooth muscle as well as in myenteric TH^+^ neurons (TGF-beta-1)^3^. Cultured myenteric neurons differentiate when treated with the TGF-betas^66^.

### The bone morphogenetic proteins (BMPs)

The BMPs are a subclass of the TGF-beta superfamily, and signal through the transmembrane receptor BMPR-II, which forms a heterotetrameric complex with a type I subunit (BMPR-IA or BMPR-IB). The type I subunits are phosphorylated by BMPR-II, and in turn, recruit and phosphorylate the downstream signal transduction molecules Smad1, 5, and 8. BMP-2 promotes differentiation and neurite outgrowth of embryonic mesencephalic dopaminergic neurons in culture^67^. In addition, BMP-2 and GDF5 promote nigrostriatal dopaminergic neuron growth via the BMPR-I –Smad1/5/8 signaling pathway. Expression of TH, BMPR-Ib, and BMPR-II mRNAs in the midbrain and striatum indicates developmental changes from embryonic to adult ages^68^. Double mutant mice lacking BMP-5 and BMP-7 have no post-mitotic dopaminergic neurons due to a lack of expression and immunoreactivity of Nurr1 and of TH and reduced expression of Lmx1a and Msx1/2, as well as of Ngn2 and Map2. Mice with conditional deletion of the *Smad1* ^*fl/*-^ gene in nestin-expressing cells exhibit a reduction of TH and SOX6 co-immunoreactive neurons in the SNpc at P0^69^.

In the E12–14 fetal gut, BMP-2 and BMP-4 as well as their receptors BMPR-II, R-IA, and R-IB are expressed in both ENCDC and non-ENCDC cell populations^70^. These BMPs exert differentiating effects on both neuronal and glial precursors, and regulate gangliogenesis^71^. They also regulate a diversity of enteric neuronal phenotypes, such as the dopaminergic subset, in which they increase the dependency on neurotrophin NTF3 (NT-3) for survival^8,70^. Transgenic mice with increased neuronal BMP-4 signaling exhibit an increased proportion of enteric TH-expressing neurons; conversely transgenic mice with decreased neuronal BMP-4 signaling (overexpressing the BMP antagonist noggin) have fewer TH- and DAT-expressing enteric neurons^8^. Furthermore, mice with a HIPK2 gene deletion, which results in excessive BMP signaling, exhibit a progressive postnatal decline in the proportion of dopaminergic neurons in both plexuses (SMP > MyP). Consequently, GI transit time is prolonged in the *Hipk2*^−/−^ mice and is accompanied by constipation with fewer and drier stool pellets than wild type^9^. A comparison of the TGF-beta/BMP signaling identified to promote differentiation, survival, and maintenance of dopaminergic neurons in the midbrain and ENS is summarized in Table 2.

**Table 2:** Compared TGF-beta and BMP signaling in the differentiation survival and maintenance of dopaminergic neurons in the midbrain and the enteric nervous system.

Midbrain-substantia nigra dopaminergic neurons			Enteric dopaminergic neurons		
Growth factors, receptors	Function	Refs	Growth factors, receptors	Function	Refs
TGF-beta-2, -3, TGF-beta-RI, -RII	Induction and maintenance of TH-IR neurons	^61^	TGF-beta-1,-2	Differentiation of MyP neurons (human colon)	^66^
Cooperate with Shh and FGF8		^62,84^	TGF-beta-R1	Expressed in TH neurons of MyP (mouse)	^3^
TGF-beta cooperates with GDNF	Survival and protection			ND in DAergic neurons	
BMP-2, GDF5	Differentiation, Increased neurite outgrowth	^67^	BMP-2, BMP-4	Increase differentiation and pSmad-1 in neurons and smooth muscle	^70^
BMPR-Ib, BMPR-II Smad1/5/8		^68^	BMPR-II, R-Ia, R-Ib	In culture	
BMP-5^−/−^,BMP-7^−/−^ double mutant mice	Absence of post-mitotic TH neurons	^69^	BMP-4	Increase dependency of TH-IR neurons on NT-3 for survival	^71^
Smad1^−/−^ conditional gene deletion	Decrease of TH- and Sox6- co IR neurons			Enhances N-CAM polysialylation and gangliogenesis, overexpressing mice	
			NSE-BMP-4	Increase NTRK3 and TH-expressing subset	^8^
			NSE-noggin (BMP antagonist)	Decrease TH- or DAT-IR neurons	
HIPK2 transcriptional co-factor, interacts with Smad1/4	Regulation of TGFbeta3 and BMP signaling	^64^	HIPK2^−/−^	Postnatal neuronal hypoplasia in MyP and SMP	^9^
HIPK2^−/−^ mutant mice	Selective loss of dopaminergic neurons	^65^		Severe loss of DA-IR and TH-IR neurons in the SMP	

### Neurotrophin-3 and brain derived neurotrophic factor

NT-3 and BDNF belong to a family of growth factors that regulate neuronal development of the CNS and the PNS. Each binds to the pan-neurotrophin binding receptor p75^NTR^/NgfR. NT-3 also binds to the receptor tyrosine kinase Ntrk3 (TrkC) and BDNF to its receptor Ntrk2 (TrkB). Both neurotrophins are highly co-expressed with TH in dopaminergic neurons of both the SNpc and the VTA^72,73^. Because all dopaminergic mesencephalic neurons in the adults express Ntrk3 and/or Ntrk2 they respond to these neurotrophins in both a paracrine and autocrine manner^74^. Mice lacking BDNF have a normal number of nigrostriatal neurons but the TH-expressing neurons have smaller dendritic arbors and migrate aberrantly to the pars reticulata rather than to the pars compacta^75^.

At E16, ENCDCs co-express Foxa2, the dopaminergic fate-determining TF, along with p75^NTR^/NgfR, indicating that precursors of newborn enteric dopaminergic neurons are already identifiable^57,76^. The markers TH, DA, DAT, and the autoreceptor D2 are co-expressed in developing enteric neurons in culture as well as in adult neurons in vivo^8,57,77,78^. Subsets of neurons in both the MyP and SMP retrogradely transport NT-3. Mice with NT-3 or Ntrk3 gene deletions exhibit severe neuronal hypoplasia, particularly in the SMP^79^. In the postnatal human ENS, BDNF is expressed in developing ganglion cells and TrkB–IR is localized to both neurons and glia^80^. Expression of NT-3 and BDNF persists in many regions of the adult GI tract including longitudinal and circular smooth muscle cells and becomes altered in dextran sodium sulfate (DSS)-induced colitis in rats^81^.

### The neurotransmitter serotonin

(5-HT) has been shown to influence the midbrain dopaminergic system via a major projection of serotonergic neurons from the dorsal raphe nucleus to the SNpc. These two neural systems interact with each other during development as well as functionally in the adult brain^82^.

Treatment of cultured ENCDC with 5-HT resulted in an increase in total differentiating as well as TH-expressing neurons demonstrating an overall neurotrophic effect of 5-HT^77^. In the ENS, neuronal serotonin is synthesized by the tryptophan hydroxylase-2 (TPH2) and expressed by serotonergic neurons, which only reside in the MyP. Terminals immunoreactive for the serotonin transporter (SERT) have been shown to surround the cytoplasm of dopaminergic neurons^77^. Mice lacking *TPH2* exhibit hypoplasia of dopaminergic neurons in the MyP with longer GI transit time but accelerated gastric emptying. The DA D2 receptor (DrD2) is expressed on subsets of both MyP and SMP neurons and mice with a Drd2 gene deletion exhibit accelerated GI motility. These results suggest that GI motility is dysregulated when an imbalance of serotonergic versus dopaminergic signaling occurs. In contrast to the growth factors described above that are mostly characterized as developmental factors, GDNF and cerebral dopamine neurotrophic factor (CDNF) have also been investigated for protective roles in the adult midbrain and on enteric dopaminergic neurons.

### The glial cell-derived neurotrophic factor (GDNF) and related family members

The neurotrophic ligands GDNF, neurturin, artemin, and persephin bind to the transmembrane signaling receptor tyrosine kinase RET. The RET-bound ligands form a tripartite complex with one of the four glycosyl phosphatidylinositol–anchored membrane co-receptors (GFR alpha subtypes 1–4), each being ligand specific. For example, GFR alpha-1 is specific for GDNF and GFR alpha-2 is specific for neurturin. The GDNF family members play essential roles in midbrain dopaminergic neuron differentiation and maintenance. Importantly, GDNF cooperates with TGF-beta to promote survival and protection of midbrain dopaminergic neurons^83^. Clinical trials assessing a potential protective and regenerative role for GDNF on the nigrostriatal system in Parkinson’s patients, however, have been inconclusive^84^.

Mice with GDNF gene deletion lack an ENS^46,85^. GDNF stimulates the proliferation of enteric neuronal precursors and their neuronal differentiation, increases the expression of its co-receptors GFR-alpha-1 and GFR-alpha-2, thus allowing their functional interaction with RET and elevating the number of neurons expressing NTRK3^86^. Notably, treatment of cultured ENCDC with GDNF significantly increases the density and proportion of dopaminergic neurons compared to controls^57^. A recent paper reports the conserved role of BMP-2 and its ortholog BMP-2b in ENS development in zebrafish and how it regulates the expression of GDNF. This study also documents decreases in protein levels of both BMP-2 and GDNF in gut samples of patients with Hirschsprung’s disease. The study confirmed via quantitative PCR that BMP-2 treatment of cultured neural crest cells increased the expression of TH^87^. A comparison of neurotrophic factors signaling pathways that promote the differentiation, survival, and maintenance of dopaminergic neurons in the midbrain and/or ENS is summarized in Table 3

**Table 3:** Compared neurotrophic factors signaling in the differentiation survival and maintenance of dopaminergic neurons in the midbrain and the enteric nervous system.

Midbrain-substantia nigra dopaminergic neurons			Enteric dopaminergic neurons		
Growth factors, receptors	Function	Refs	Growth factors, receptors	Function	Refs
NTF3, NTRK3/trkC	Development and maintenance in rats and mice	^3,7,72–74^	NgfR/P75^NTR^	Pan ENCDC marker	^57,76^
			NTF3, NTRK3 (rats and mice)	Survival, differentiation MyP, and SMP	^79^
			NTF3^−/−^ NTRK3^−/−^	Neuronal hypoplasia SMP>MyP including DA neurons	^8^
			NTF3 and BMP-2	Increase DA neurons	
BDNF,	Expressed in dopaminergic neurons of SN	^72^	BDNF	Expressed in human postnatal ENS development and survival	^80^
NTRK2/trkB	Reduced dendritic maturation	^74^			
BDNF^−/−^	Aberrant location of TH-IR cell bodies	^75^	NTRK2/trkB	Not Det. for DA neurons	
5-HT/serotonin	Interaction with midbrain dopaminergic neurons in development and adulthood	^82^	5-HT	Differentiation from ENCDC into DA neurons	^77^
			TPH2^−/−^ (loss of neuronal 5-HT)	Decreased density in MyP Not Det. in SMP	
GDNF, Neurturin, Persephin, Artemin	Essential roles in differentiation and maintenance	^84^	GDNF, Ret	Nestin-IR proliferation, then neuronal, not glial, differentiation	^86^
			GFR alpha-1, GFR alpha-2	Increased GFRα1 and TrkC	

### The cerebral dopamine neurotrophic factor (CDNF) and mesencephalic astrocyte neurotrophic factor (MANF)

Recently, a new family of evolutionarily conserved neurotrophic factors was identified, including CDNF and MANF/Arginin-Rich Mutated Early stage Tumors (ARMET). ARMET/MANF was originally identified as a factor released from rat astrocytes of ventral mesencephalon that supported the survival of cultured rat embryonic dopaminergic neurons. It is highly conserved across evolution, present in *Caenorhabditis elegans*, *Drosophila melanogaster*, rodents, and humans. Similar to CDNF, it exerts protective effects on SN dopaminergic neurons under ER stress^88^.

CDNF has been localized to postnatal and adult nigrostriatal axons using immunocytochemistry^89^ but it can also be expressed by neurons other than the dopaminergic subset^88^. In contrast to other neurotrophic factors, CDNF has a dual mode of action. As a mostly resident protein within the ER, it binds via its C terminal domain to proteins involved in the regulation of ER stress and the unfolded protein response (UPR). Its other mode of action is like that of other neurotrophins, acting as a secreted protein that can bind via its N terminal domain to membrane lipids and activate still unidentified transmembrane receptors^90^. CDNF is localized to the substantia nigra in postnatal mouse brains^89^. Mice with a CDNF gene deletion (Cdnf^−/−^) exhibit unaltered survival of dopaminergic neurons in the SN or of striatal DA level^91^. Thus, while CDNF is dispensable for the maintenance of healthy nigral dopaminergic neurons, it does strongly protect and restore these same adult neurons following intrastriatal injection of 6-OHDA in the rat model of PD^89^.

CDNF has also been investigated for its role in the development and long-term survival of enteric dopaminergic neurons. CDNF expression is restricted to subsets of enteric neurons in both plexuses. In the SMP, it is expressed by 76% of dopaminergic neurons, which constitute only 25% of all the CDNF-immunoreactive enteric neurons^57^. In cultures of enteric progenitors (ENCDC), CDNF selectively promoted differentiation of neurons co-labeled with TH and Foxa2, but did not affect total neuron survival. Pre-exposure to BMPs, however, synergistically enhanced the CDNF-promoted differentiation of enteric dopaminergic neurons and induced their dependence on CDNF for survival^57^. It is thus conceivable that this exacerbated response to CDNF could be due to an ER-stress response induced by BMP on the differentiating dopaminergic neurons precursors, as has been demonstrated to occur in osteoblasts undergoing BMP differentiation^92^. CDNF was more selective than 5-HT in promoting the development of enteric dopaminergic neurons, but less effective than GDNF. The combination of CDNF with GDNF had no additive effects. CDNF interferes with ER stress levels while GDNF or 5-HT do not, and conversely, these two molecules promote neurogenesis while CDNF does not. Thus, in developing dopaminergic neurons CDNF can activate downstream signaling pathways that are different from those activated by either GDNF or 5-HT^57^. A recent study performed in zebrafish analyzed the impact of *Cdnf* loss from larva to adult stages on the development of several neuronal subtypes, including dopaminergic neurons. Due to gene duplication, there are two alleles of TH in zebrafish, Th1, and Th2. In the *Cdnf* mutant, Th2 expression was selectively upregulated and there was an increase in the number of TH2-IR neurons. Nevertheless, the levels of DA in Cdnf-null zebrafish were comparable to those of wildtype controls throughout the brain, from the larval stages through late adulthood. The numbers of GABAergic neurons in the hypothalamus of the mutant zebrafish larvae were reduced, similar to the reduction in enteric neurons expressing GABA in the SMP of *Cdnf*^−/−^ mice^93^. From this evidence, it is clear that as a highly evolutionarily conserved factor, CDNF can exert different roles (development, maintenance, protection) depending on the type of neurons it impacts and on the species in which it is expressed. A comparison of neuroprotective factors that promote the differentiation, survival, and maintenance of dopaminergic neurons in the midbrain and/or ENS is summarized in Table 4.

**Table 4:** Compared neuroprotective factors signaling in the differentiation survival and maintenance of dopaminergic neurons in the midbrain and the enteric nervous system.

Midbrain-substantia nigra dopaminergic neurons			Enteric dopaminergic neurons		
CDNF	Function	Refs	Growth factors, receptors	Function	Refs
CDNF	Regulation of ER stress and the UPR	^89^	CDNF	Only expressed in neurons of MyP and SMP	^57^
	Does not promote survival or differentiation			In majority of DA neurons	
				Increases density and % of DA neurons from ENCDC	
			CDNF+GDNF	Proportion of differentiating DA neurons, enhanced	
CDNF^−/−^	No loss of dopaminergic neurons in the SN	^91^	CDNF+ BMP-2- or -4	DA neurons become dependent on CDNF for survival	^57^
			CDNF^−/−^	Decreased DAT expression, deficit of neurons only in SMP, DA neurons most severely affected	

By postnatal days P17-P21 in the rat, differentiated dopaminergic nigral neurons exhibit elongated axons and establish synaptic connections with specific phenotypes of target neurons within the striatum^94^. In the murine ENS, it is likely that connections between myenteric and submucosal dopaminergic neurons and their postsynaptic target neurons are established by post-weaning ages (P21).

## Dopaminergic neurons provide modulatory inputs to their postsynaptic targets and exhibit sustained excitability in the SNpc and in the ENS

### Midbrain

The dopaminergic neuronal input from the SNpc to the striatum can result in either net excitation or inhibition of the postsynaptic neurons, depending on the subtype of the targeted DA receptor. For instance, SNpc dopaminergic neurons can enhance the activity of cholinergic interneurons in the striatum via D1/D5 receptors or inhibit them via D2/D3/D4 receptors. In turn, nicotinic receptors on dopaminergic axons and cell bodies can enhance or depress presynaptic DA release^95,96^. As an added complexity, the amount of DA released can also be highly modulated presynaptically at axons^97^. Striatal gabaergic neurons can inhibit the release of DA via activation of Gaba_A_ and Gaba_B_ receptors expressed on dopaminergic axons^98^. Because SNpc neurons receive cholinergic input from striatal cholinergic interneurons as well as from the pedunculopontine and laterodorsal tegmental nuclei and express presynaptic nicotinic and muscarinic receptors, ACh can also modulate the release of DA from the nigral neurons^99^.

Another tier of functional modulation of dopamine nigral input is modulation of the activity of the cholinergic interneurons of the striatum. Through the use of optogenetic stimulation, slice electrophysiology, retrograde tracer injection, and pharmacological manipulations, a subset of SN neurons that project to the lateral dorsal striatum have been revealed to exert two effects on cholinergic interneurons, first from DA release and subsequently in response to glutamate release from the same neurons^100^. These neurons, via D2 receptors, first inhibit the cholinergic interneurons, and then due to their co-release of glutamate, promote a slow excitation via mGlur1 and partially via D1-like receptors coupled to transient receptor potential channels 3 and 7.

Dopaminergic neurons exhibit both sustained pacemaking and bursting activity and are highly diverse functionally. Extensive electrophysiological recordings from single nigral neurons combined with retrograde tracing and neurohistochemistry using phenotype markers have allowed the mapping of specific dopaminergic axonal projections to specific targeted areas in the striatum^101^. While neurons in the lateral and some in the medial SN project to the dorsal lateral striatum (DLS), other neurons in the medial SN project either to the lateral nucleus accumbens or the dorsal medial striatum (DMS). Notably, the SN neurons projecting to the DLS exhibit higher excitability and enhanced bursting compared to those projecting to the DMS, and are the ones selectively affected in PD^102,103^.

### ENS

**Fig. 1:**
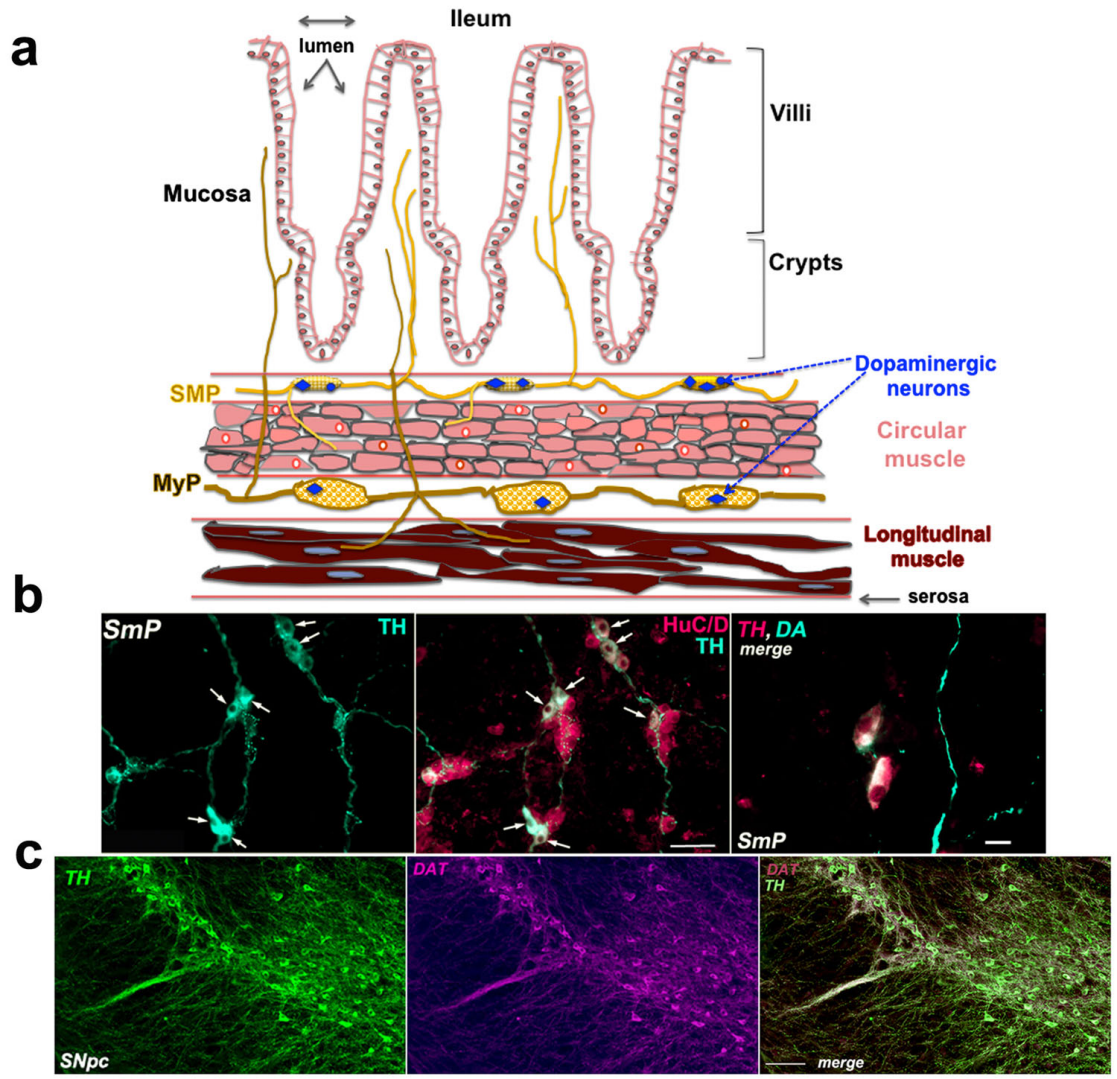
Contrasting architecture of enteric dopaminergic neurons within enteric ganglia that are organized into two plexuses and of nigral dopaminergic neurons within layers of the SNpc. **a** Longitudinal section of gut through the ileum encompassing from the lumen to the serosa: the mucosal epithelial layer with villi and crypts, the submucosal layer with the ganglionic chains of submucosal (SMP) neurons (yellow), the layers (in cross-section) of circular smooth muscle fibers (pink, brick-shaped), the ganglionic chains of myenteric (MyP) neurons (brownish yellow), the layers of longitudinal smooth muscles (dark red). Dopaminergic neurons within the ganglia of both plexuses are highlighted (blue stars). Note how the chains of submucosal ganglia are contiguous with the mucosa. **b** Fluorescence immunocytochemistry of representative whole mounts of SmP from adult mouse, displaying the dopaminergic neurons (left panel, identified with TH antibodies, green fluorescence, white arrows) and as a subset (middle panel, merged image, white arrows) of all the neurons identified with HuC/D antibodies, (magenta fluorescence); calibration bar: 70 microns. Right panel: magnified view of two dopaminergic neurons identified with both TH and DA antibodies (merged magenta and green fluorescences); calibration bar: 16 microns. This image is provided by Dr Alcmène Chalazonitis. **c** Fluorescence immunocytochemistry of a representative section of adult mouse midbrain through the Substantia Nigra with dopaminergic neurons identified with TH antibodies (left panel, green fluorescence), DAT (middle panel, magenta fluorescence) and merged image (right panel, green and magenta fluorescences). Calibration bar: 100 microns. This image is provided by Benjamin Hobson from Dr David Sulzer’s lab.

Expression of the dopaminergic phenotype in the mouse ENS is variable depending on the region of the gut. Transcripts encoding TH are higher in the stomach and Ileum than in the colon. The density of TH-immunoreactive neurons within the plexuses also varies depending on the strain. In adult CD-1 mice, the proportion of TH-IR neurons, which are also DAT-IR is relatively higher in the ileal SMP (13 ± 1%) than in the MyP (9 ± 3%)^4^. In a study in which neuronal phenotypes were analyzed in the ileums of BalbC mice, a much lower proportion of TH-expressing neurons (<0.5%) was reported in the MyP compared to that in the MyP of CD-1 mice^104^. Interestingly, in the C57BL6 adult ileum, TH-IR neurons were a minority of SMP neurons and all were co-immunoreactive with VIP^105^. In adult mice with a mixed background including C57BL6 and CD-1, the density of dopaminergic neurons expressed per mm^2^ of plexus area in the ileum, was 13.3 ± 0.5 in the SMP and only 3.5 ± 0.6 in the MyP^57^. Longitudinal sections in the ileum and colon displaying the ganglionic chains of myenteric and SMP with the highlighted dopaminergic neurons cell bodies are illustrated in [Fig. 1].

The intrinsic presynaptic neuronal input to enteric dopaminergic neurons as well as their postsynaptic neuronal targets have yet to be systematically characterized in the MyP as in the SMP. In the guinea pig stomach, some DA-immunoreactive neurons have been identified and dopaminergic immunoreactive fibers are shown to encircle choline acetyl transferase immunoreactive neurons in the MyP. Blockade of D2 receptors moreover potentiates the release of acetylcholine, implying an inhibitory action by enteric DA^106^. More recently SERT-immunoreactive (putatively serotonergic) terminal axons have been shown to encircle dopaminergic neurons in the MyP of the ileum^77^. These neuronal connections were identified in the MyP, but have not been analyzed in the SMP, although it is the plexus where enteric dopaminergic neurons are most abundant^4,57^.

A more comprehensive understanding of the function of dopaminergic neurons in the ENS has resulted from assessing GI motility in mice with genetic deletions affecting dopaminergic neuron development, DA reuptake, or DA receptors. Colonic motility assessed in an ex vivo organ bath system exhibits increased tone and phasic spontaneous or elicited contraction amplitudes in WT mice when D1 and D2 receptors are blocked. While mice with deletion of the DAT gene (*DAT*^−/−^) exhibit a lower degree of increased spontaneous or elicited contraction compared with WT mice, following D1 and D2 receptor blockade, these mice displayed enhanced phasic and elicited contractions similar to those in WT mice. Thus, neuronal dopamine exerts a brake on distal colonic motility^107^. Mice with a D2 receptor gene deletion exhibit accelerated GITT and colonic expulsion time as well as a compensatory increase in expression of TH and DAT transcripts, suggesting that DA neurons may exert an overall inhibition of GI motility^77,78^. In contrast, mice with the *Cdnf* gene deletion that exhibits an age-exacerbating hypoplasia of dopaminergic neurons and other late-born neurons in the SMP have longer GITT, less efficient gastric emptying, and slower colonic expulsion time^57^, implying that GI motility is affected differently. This may occur because the dopaminergic neurons are dysfunctional or because they interact with other late-born degenerating neurons.

Overall, these GI motility analyses are the end result of interference with dopaminergic function and do not shed light on defining the precise neuronal networks involved. Several types of neurons including enteric motorneurons, interneurons, intrinsic primary afferent neurons, and secretomotor neurons expressing a combination of neurotransmitters and neuropeptides and exerting either excitatory or inhibitory input were identified to control peristalsis and secretion in the guinea pig. However, at the time of such work, data on dopaminergic neurons were not available^108^. Considering the chains of ganglia in each MyP and SMP and that both plexuses are interconnected^109^, it is likely that during peristalsis, repeated patterns of networks are activated in a caudal and/or rostral fashion. These networks may differ depending on GI tract region in order to control their specific functions. Recent reports using optogenetic stimulation in transgenic mice have revealed which phenotypes of neurons are involved in controlling the propagation of the colonic migrating motor complex (CMMC). Cholinergic neurons expressing GCaMP (ChAT-GCaMP) in the MyP display increased activation during the CMMC, while nitrergic neurons expressing GCaMP (nNOs-GCaMP) display increased activity during tonic inhibition of the CMMC. Furthermore, optogenetic activation of channel rhodopsin-expressing nitrergic neurons inhibits the ongoing CMMC and exposure to a NO synthesis inhibitor exacerbates the activity of ChAT-GCaMP neurons^110^.

Dynamic changes of Ca^2+^ uptake, traceable via high-resolution imaging in myenteric neurons, combined with extracellular or intracellular recordings of the underlying innervated smooth muscle in the murine colon, have beautifully demonstrated that generation and coordination of the CMMCs are consequent to the rhythmic and synchronized firing of populations of myenteric neurons located in different ganglia and propagated along the colon. During prolonged neurogenic firing bursts, large numbers of cholinergic and nitrergic myenteric neurons are synchronously active as well as CGRP-expressing intrinsic primary sensory neurons, resulting in the contraction of smooth muscles at about 2 Hz frequency^111,112^. Thus, use of mice genetically modified to express fluorescent proteins (i.e., GFP or channel rhodopsin2 or GCaMP3) on promoters of genes specifically expressed by dopaminergic neurons (TH, DAT, Drd2, Drd3) could identify them and define their function within the ganglionic plexuses. Immunohistochemistry using antibodies specific for many of the known enteric neuronal phenotypes and optogenetic stimulation could further reveal which neuronal phenotype(s) exert presynaptic input onto the dopaminergic neurons and which neuronal phenotype(s) are the postsynaptic target(s) of the dopaminergic neurons and whether these neuronal targets are activated or inhibited.

As dopaminergic neurons age, they are susceptible to degeneration, as particularly observed for the SNpc neurons in PD and related parkinsonism. As only a minority of older people develops these disorders, there must be “multiple hits” that in addition to aging make them vulnerable.

## Intrinsic and extrinsic vulnerability of midbrain and enteric dopaminergic neurons may explain their targeting in PD

### Midbrain

Compared to other types of brain neurons, the midbrain dopaminergic neurons located in the SNpc exhibit an intrinsic vulnerability that becomes manifested in the adult in PD^102,113^. We note however that their progenitors, which arise from the median FP, require an expression of distinct TFs for their migration (Sox6), and for terminal differentiation, long-term maintenance, and aging (Pitx3) that are not expressed by the progenitors of less vulnerable dopaminergic neurons^114^. The combined deletion of Foxa1 and Foxa2 results in a complete loss of neuronal DAT with a reduction of SN dopaminergic neurons and locomotor deficits reminiscent of PD, a downregulation of TH and dopamine biosynthesis in the SNpc, with reduction of burst firing activity of the nigral neurons and hence of neurotransmission in the striatum^115^. Interestingly, these mice exhibited pronounced feeding behavior deficits^116^. Thus both Foxa1 and Foxa2 are required for the maintenance of adult dopaminergic neurons and their function. Nurr1 and En-1 are not only necessary for the survival and differentiation of mesencephalic dopaminergic neurons, but have been shown to be critical for the long-term survival and protection of these neurons against mitochondrial dysfunction^117^. Another TF Pitx3 can protect adult and aging dopaminergic neurons specifically located in the ventral SN, which exhibit an increased vulnerability to MPTP exposure^118^. The TFs Lmx1a and Lmx1b are involved not only in the fate determination of dopaminergic neurons progenitors but also control survival of adult midbrain dopaminergic via prevention of mitochondrial dysfunction and alpha-synuclein aggregation^119^.

As of this writing, more than 20 genes and risk factors for PD have been identified, many of which are associated with autophagy and lysosomes, mitochondria, the cytoskeleton, and changes in the immune response. The majority of PD cases are still “idiopathic” in that their etiology is unknown, although virtually all PD patients show the presence of Lewy bodies, which are formed in large part of aggregated alpha-synuclein, a presynaptic protein that regulates DA release by synaptic vesicle exocytosis^120^. It is widely suspected that pathogenesis of PD entails molecular deregulation that leads to α-synuclein misfolding and aggregation, Lewy body formation, an apparent spread from neuron to neuron, and consequent neurodegeneration of multiple neurons, but particularly the neuromelanin-containing neurons of the SNpc and locus coeruleus^113,121–123^. There have been many suggestions for why dopaminergic neurons die in PD, some of which encompass combinatorial multiple “hits” that include aspects of aging^124^. Some hypotheses include roles for the presence of DA itself, which is highly reactive and when oxidized within the cytosol leads to the production of neuromelanin in autophagic vacuoles^125^. In addition, oxidation of cytosolic DA is associated with disruption of normal mitochondrial and protein turnover, particularly by autophagic processes that become less efficient during older age^126^. Furthermore, reactions of oxidized DA with alpha-synuclein^127,128^ may further block autophagy^129^ and lead to the production of neoantigens that may promote features of autoimmunity^130^. At this point, it seems likely that there are many different types of pathogenic pathways that lead to PD, but that they converge on disruption of normal turnover of α-synuclein, leading to Lewy bodies, and the death of pigmented neurons of the SNpc and locus coeruleus.

### ENS

As is the brain, the ENS is a prominent target of neurodegenerative diseases including PD^13^. Increasing evidence demonstrates an interaction between the gut and the midbrain in the etiology of PD. Colonization of the gut of αlpha-synuclein overexpressing/ASO mice with microbiota from PD patients exacerbates their motor deficit, compared to the gut of mice colonized with microbiota of healthy donors^131^. In the *Pink1*^−/−^ gene deletion model, mice develop mitochondria-specific cytotoxic CD8^+^T cells. When their intestine is infected with Gram-negative bacteria, the mice then exhibit motor impairment and decreased axonal dopaminergic varicosities in the striatum^132^. Moreover, independent studies of very large populations have shown that there is an increased incidence of PD in subjects with an inflammatory bowel disorder, and that such subjects who have been treated with anti-TNF-alpha therapy have a substantially decreased probability of developing PD^133^. Another link between damage in the SN and the ENS has been demonstrated in a rat model following nigral injection of lipopolysaccharides (LPS). The ensuing response to LPS triggers inflammatory cytokines which then promote degeneration of the dopaminergic neurons, and which gets exacerbated when ulcerative colitis is induced in the rats by DSS treatment^134^. The several ways in which the ENS, the mucosa, and gut function are primary targets of intrinsic pathological αlpha−synuclein and its spread have been described^135^. A recent extensive review cites evidence of accumulation of alpha-synuclein due to GI inflammation and immune system response and describes an understanding of the context of why patients with ulcerative colitis exhibit increased risk of PD^136^. The degree of severity of the disease in PD patients was shown early on to correlate directly with abnormal salivation, dysphagia, nausea, and defecation dysfunction, demonstrating a direct association with the GI tract^137^. Recent sequencing analysis at the single-cell resolution level performed on the human and mouse ENSs has demonstrated the expression of PD susceptibility genes in the neurons of the colon, including *DLG2*, *SNCA*, and *SCN3A*, with the latter validated in situ by immunocytochemistry^11^.

Aging rodents and humans display neurodegeneration in the ENS^138,139^. To determine whether enteric dopaminergic neurons could be a specific target of degeneration in a mouse model of PD, mice were injected intraperitoneally with MPTP. Ten days later immunocytochemistry analyses in the toxin-treated mice compared to saline-treated mice indicated a 40% reduction in TH-labeled neurons in the MyP. In contrast, nitrergic and cholinergic neuron densities were not affected. Circular muscles exhibited significantly impaired neural-mediated relaxation and transient increases in colon motility. The SMP was not analyzed^140^. However, the neurons of the SMP are more vulnerable compared to myenteric neurons due to the lack of a blood barrier. Such a barrier limits the access of intravascular macromolecules to the MyP and muscularis externa, but not to the SMP^141^. As an example, a reduction in the number of neurons in the SMP of the colon is exacerbated with age in Fischer 344 rats with swollen TH-immunoreactive axons and terminals^142^. In the murine ileum, both myenteric and submucosal neuron densities also decline with age. Some of the aging dopaminergic neurons in the MyP also display aggregation of alpha-synuclein immunoreactivity^143^. To model familial forms of PD a transgenic mouse model was developed to express the human PD-associated α−SYNC gene mutation A53T; such animals display early GI abnormalities as those occurring in PD patients. Most TH-labeled neurons in the SMP of the ileum are co-immunoreactive with alpha-synuclein^144^. Early manifestations of PD symptoms affect organs that are exposed to toxic insults such as the ENS proximal to the gut lumen. Misfolding and aggregation of alpha−synuclein and Lewy Body pathology have been localized to the gastric myenteric and SMPs and it was hypothesized early on by H. Braak to spread via transynaptic retrograde propagation from vulnerable neurons to the dorsal motor nucleus of the vagus (dmX) in PD patients^145^. Recent reports have confirmed such a propagation mechanism. Inoculation of α-synuclein preformed fibrils (PFF) into the gastric wall of a mouse promoted a retrograde accumulation of Lewy body like-aggregates in the dmX that interestingly, declined 45 days post inoculation. The accumulation in the dmX did not occur when the inoculation of alpha-synuclein PFF was performed following vagotomy. Because the retrograde transport of alpha-synuclein aggregates via the vagus nerve was transient in this model, other mechanisms have to be invoked to promote the persistence of the aggregates in the dmX^146^.

Consistent with the consequences of higher exposure of submucosal neurons to toxic insults, colon biopsies examined by an international consortium of pathology laboratories from a large number of Parkinson’s patients (age range 77.4) demonstrated that the highest prevalence of pathological Lewy type α-synuclein accumulation occurs primarily in the submucosa followed by the muscularis and then the mucosa^147^. Support for Braak’s hypothesis has been recently demonstrated in a new mouse model in which pathological alpha-synuclein (pSer129 alpha-synuclein) is inoculated in the smooth muscles of the pylorus and duodenum, the most highly innervated gut region by the vagus. By 1 month post inoculation, enteric neurons exhibit pSer129 alpha-synuclein immunoreactivity. However, it is not until 7 months post inoculation that the number of dopaminergic neurons in the SNpc and levels of TH and DAT in the midbrain decline^148^. Another recent report has confirmed that one month following alpha-synuclein PFF inoculation in the duodenum of aged mice, alpha-synuclein PFFs are present in myenteric neurons, promoting gut dysfunction via impaired synaptic transmission within the ENS^149^. Notably, the retrograde propagation of α-synuclein PFF to the midbrain is more significant in the DMV than in the SNpc. An additional factor to be considered is the lysosomal enzyme, glucocerebrosidase (GCase), which promotes pathogen clearance and decreases α-synuclein aggregates. In Gaucher’s disease both copies of the gene encoding GCase, *GBA1*, are mutated and α−synuclein accumulation is increased in the brain. With aging, GCase clearance capacity declines. In mice constitutively overproducing α-synuclein (ASO mice), GCase can be over-expressed via *GBA1* gene transfer selectively in the PNS using the AAV-PHP.S vector system. Such systemic delivery can reinstate GCase production, and reduces α-synuclein accumulation in the duodenum of ASO mice. Moreover, the ENS of ASO mice with over-expressed *GBA1* display restored enteric neuronal network activity as recorded via optogenetics stimulation^149^. In this study, the specific ENS phenotypes that were rescued were not identified.

At this juncture, the question of whether dopaminergic neurons are the most likely phenotype targeted to degenerate and die in the ENS of PD patients is unsettled. One report shows no loss of dopaminergic neurons or of noradrenergic innervation in the SMP in colonic biopsies of live PD patients (mean age 62.8 ± 5)^150^. Recently integrated genome-wide association studies and single-cell transcriptome data derived from multiple central and PNS samples demonstrate a significant genetic association of PD not only with SNc neurons, but also with enteric neurons^151^. An approach geared at detecting the early manifestation of PD, prior to aggregation of alpha-synuclein, was used to measure Ca^++^ currents and mitochondrial potential in neurons of the duodenal SMP. The study did not find differences between neurons from PD patients with those of their healthy partners^152^. However, there are many caveats to using this study to conclude that there is a lack of damage to ENS dopaminergic neurons in PD. These include that the phenotypes of the recorded SMP neurons were not identified. Since the biopsies were mostly from patients of younger age (mean age 58.9 ± 9) there is a possibility that the recorded neurons could have recovered or that they are located in a protected region of the duodenal SMP: we note that biopsies only measure small regions of accessible ENS tissue. In contrast, recent analyses of biopsies of human jejunum and colon isolated from deceased PD patients (age range 73–84; mean age 77) present evidence of atrophied degenerating neurons in both MyP and SMP with colocalized alpha-synuclein deposits, although whether those are dopaminergic was not examined^153^. Comparing the analyses of biopsies from live younger patients with those from older deceased patients suggests that aging exacerbates the evolving impact of PD on submucosal neurons, although it may be that the damage or death of dopaminergic neurons occurs in inaccessible regions to biopsy, such as the ileum. Recently a multiplexed mRNA profiling analysis was carried out from deep submucosal rectal biopsies of PD patients and healthy controls (mean age 65) to compare the expression profile of genes involved in neuropathological processes. Biopsies from the majority of PD patients displayed altered expression of 22 genes involved in neuroglial functions, mitochondrial function, vesicle trafficking, and inflammation. Involvement of some of these genes within enteric neurons was demonstrated via immunohistochemistry for the pan neuronal marker (PGP9.5). Again, no specific neuronal phenotype markers were analyzed^154^.

## Identification of neuroprotective signaling for midbrain and enteric dopaminergic neurons exposed to toxic insult, degeneration, and/or aging

### Midbrain

Beyond their development, the long-term maintenance of midbrain dopaminergic neurons can be supported by several neurotrophic and neuroprotective signaling as reviewed early on^5^ and more recently in clinical trials on human patients^155^. Various approaches have yielded contradicting results regarding the protective effects of the BMPs. In one study using the neurotoxin 6-OHDA-treated rat model, no protection by either BMPs or GDFs occurred on the dopaminergic neurons to prevent motor impairment^156,157^. Another study using the same model however reported that intranigral delivery of BMP-7 prevented nigral hypoplasia, restored DA release in the striatum, and reduced motor deficits^158^. In a follow-up study, impaired BMP signaling in adult male mice expressing a dominant negative BMPR-II receptor (BMPRIIDN) resulted in a loss of TH-labeled neurons in the SNpc with decreased neurite fiber density of the SN reticulata accompanied by impaired locomotor activity^159^. When BMPRIIDN mice were exposed to high doses of methamphetamine, which leads to loss of the neurites of dopaminergic neurons, increased cell death occurred in the SNpc compared to that observed in WT mice. These results demonstrate the trophic and protective effects of BMPs on nigrostriatal pathways. Because endogenous BMPs promote the survival of midbrain dopaminergic neurons, they are considered potential protective factors against degeneration in PD^160^.

Another signaling pathway that can be interfered with to provide protection of nigral dopaminergic neurons is that of the nerve growth factor (NGF) precursor, proNGF. ProNGF binds with high affinity to the p75^NTR^ receptor to form a complex with the co-receptor sortilin, and the complex can promote cell death. Because this complex is found in dopaminergic neurons of the ventral SN, it could exacerbate hypoplasia and dysfunction of these neurons as in PD, and interfering with this complex could thus be potentially beneficial in therapy against PD^161^.

The primary treatment for PD is Levo-dopa, which exerts temporary benefits in patients who eventually exhibit dyskinesia^162^. Cell replacement therapy provides an approach to treat PD and the means by which human pluripotent stem cells may be converted into mesencephalic dopaminergic-like neurons are in development^163^.

Other approaches employ treatment with neurotrophic factors. GDNF and neurturin were the first neurotrophic factors tested as therapies using a partial lesion model of PD^164^. Subsequently, GDNF delivered via intermittent injection over an extended period into the putamen in patients with moderate PD was found to not significantly improve symptoms^165^.

In this context, treatment with the newly identified neurotrophic and neuroprotective factor CDNF has provided an alternative approach. CDNF is expressed in the mouse and human brain including in the SN and striatum, and has been tested to evaluate whether it can counteract toxic exposure of midbrain dopaminergic neurons in mouse models of PD. CDNF rescues nigral dopaminergic neurons and reduces amphetamine-induced rotational behavior, an indicator of unilateral dopaminergic neuron depletion, following intrastriatal unilateral injection of 6-OHDA in a rat model of PD^89^. CDNF also protects the nigrostriatal DA system and promotes recovery after MPTP treatment in mice^166^. Notably, CDNF treatment has been directly shown to protect mesencephalic dopaminergic neurons in culture against toxicity induced by A30P α-synuclein oligomers^167^. In addition, the long-term maintenance role of CDNF in the function of nigral dopaminergic neurons has been demonstrated in aged Cdnf^−/−^ male mice since they exhibit aberrant DAT function, resulting in altered DA release from axons in the striatum^91^.

Patients exhibiting early-onset PD have been analyzed for possible mutations in the CDNF gene, but no mutations have been identified. However, a C allele of an intronic CDNF single nucleotide polymorphism was identified as conferring increased susceptibility to PD^168^. A comprehensive overview of the neuroprotective factors tested to date in the prevention and treatment of neurodegeneration in rodent and non-human primate models of PD, as well as in clinical trials, includes MANF and CDNF^169,170^. In contrast to other neurotrophic factors, MANF and CDNF are resident in the ER and counteract neuroinflammation-producing ER stress, which is prominent and sustained in the evolution of neuronal degeneration. A mechanism of action of CDNF has now been demonstrated via its molecular interaction with α-synuclein, and interference with the internalization of the fibrils and their spread^171^. Because of their safety parameters, CDNF and MANF are strong candidates for development as therapeutic treatments. Intraputamenal infusion of CDNF using an implanted drug delivery system monthly for 6 months is currently being performed in a phase I-II clinical trial on patients with idiopathic moderate PD.

### ENS

The role of CDNF in long-term maintenance of total and dopaminergic enteric neurons and of functional GI motility was revealed in mice with a CDNF gene deletion (*Cdnf*^−/−^)^57,91^. Considering the age-dependent evolution of PD from prodromal disease in the ENS to full motor manifestations following midbrain degeneration, *Cdnf*^−/−^ mice do not exhibit a significantly enhanced age-related decline in neuronal density in the MyP compared to that of *Cdnf*^+/+^ mice at any age examined (from 1.5- to 12-month old). In contrast, in the SMP, age-related neuronal hypoplasia is exacerbated in the 9–12-month-old *Cdnf*^−/−^ mice compared to WT littermates^91^. Notably, more than the changes in total neuronal density, the proportions of nitrergic, GABAergic and CGRP-expressing subsets are all significantly reduced, with that of the dopaminergic neurons being the most severely affected^57^. Whereas GI motility parameters in the cohort of adult *Cdnf*^−/−^ mice were not significantly different from those in adult WT mice, the total GI transit time and colonic expulsion time were significantly prolonged and the gastric emptying efficiency declined in aging adult *Cdnf*^−/−^ mice compared to aging WT. Thus CDNF is essential for maintaining the integrity and function of the dopaminergic and other late-born subsets of submucosal neurons involved in the peristaltic reflex, and thus for proper GI motility during aging^57^.

Overall, these reports demonstrate that the submucosal neurons including the dopaminergic subset are far more vulnerable than the myenteric neurons in the absence of neuroprotection by CDNF. The mechanism(s) through which the exacerbated hypoplasia of submucosal neurons occurs as early as 1.5 months in *Cdnf*^−/−^ mice does not involve apoptosis, but apparently another form of neurodegeneration. The occurrence of neurodegeneration without apoptosis is an evolutionary conserved mechanism that has been reported to occur in nigral neurons in the MPTP+ mouse model^172^ and recently in a *C. elegans* model where activation of the UPR was sustained in mitochondria^173^. Thus, the neurodegeneration occurring in the SMP of *Cdnf*^−/−^ mice implies that CDNF may be important for the postnatal maintenance of these neurons^91^.

Autophagy is a general cellular stress response, and enhanced density of autophagosomes, including elevated LC3 II-immunoreactivity, is displayed by neurons of the SMP of aging (9–12-month-old) Cdnf^−/−^ mice compared to age-matched Cdnf^+/+^ littermates. In contrast, the levels of autophagy are comparable in adult (3-month-old) Cdnf^−/−^ and Cdnf^+/+^ mice. Increased Fluoro-Jade C, a marker of neurodegeneration, is also detected selectively in the submucosal neurons in the duodenum, ileum, and colon of Cdnf^−/−^ mice^91^. With sustained stress, autophagy might eventually lead to neurodegeneration^174^. The high level of stress impacting ENS neurons is likely to be the result of continuous excitation and inhibition of large populations of myenteric and submucosal neurons that control the CMMC and gut peristalsis^112^. This functional property is reminiscent of the higher level of excitability and enhanced bursting activity exhibited by the dopaminergic neurons of the SN that project to the lateral dorsal nucleus and are selectively affected in PD^103^. The therapeutic potential of CDNF needs to be tested in clinical trials by targeting the ENS to treat patients who exhibit GI dysfunction prior to motor symptoms, and evaluating whether further propagation of alpha-synucleopathies back to the dmX can be halted.

## Concluding remarks

The midbrain/SNpc and the enteric dopaminergic neurons originate and develop from different locations, namely the mesencephalic FP for midbrain neurons and the pool of ENCDCs that colonize the fetal gut. The specification of dopaminergic precursors occurs earlier in the midbrain (E9.5 in mouse) than in the gut (E15.5–P0 in mouse). They thus become post-mitotic much earlier in the midbrain (E10.5–E12 in mouse), whereas most of the enteric dopaminergic precursors do not leave the cell cycle until E15.5 in mouse. TFs such as Phox2b, Zeb2, and Sox10 that differ from those identified in the developing SN, are involved in the control of colonization, migration, and settling of the ENCDC into ganglia. Two TFs have been identified so far that are similarly involved in the specification and commitment to the dopaminergic fate: Sox6 and Foxa2 in the maturing SN (at E9.5–18 in mouse) and in the ENCDC (at E15–E16 in mouse). By E18.5, when colonization of the mouse gut is complete, additional TFs have been identified that are expressed much earlier by SN neurons such as Ebf1, Pbx3, Ascl1,. It is remarkable that many of the growth factors and their signaling pathways involved in the subsequent differentiation, survival, and maintenance of dopaminergic neurons are comparable in the developing SN and ENS, at least until the weaning stage. Some of the TFs such as Foxa2 are also involved in the long-term maintenance of both adult neuronal populations.

However, in the adult, the two environments are very different. Whereas SN dopaminergic neurons are relatively protected by the blood–brain barrier (BBB), enteric dopaminergic neurons, particularly the subset in the SMP, lack a blood barrier and are both exposed to potentially toxic elements that may translocate across the epithelium from the gut lumen and are in close proximity to the gut microbiome. Despite the BBB, both neuronal populations exhibit a vulnerability that exacerbates with age and may be a consequence of similar patterns of sustained excitability and firing, leading to the impacts of higher stress. It is thus not surprising that in neurodegenerative diseases such as PD, alpha-synuclein acquires a pathological form that forms fibrils that aggregate and interfere with normal synaptic transmission. With time, increased density of autophagosomes, neurodegeneration, and dysfunction ensue and tend to be manifested earlier in the ENS than in the SN. At this time, most reported immunohistochemical and functional analyses of gut biopsies isolated from PD patients have not examined which neuronal phenotypes are the most affected, including the dopaminergic subset. Several potential neuroprotective and regenerative treatments involving the control of alpha-synuclein aggregation and neurotrophic molecules are being developed. In particular, CDNF (phase I-II clinical trials) and MANF are being tested as a potential therapy in the midbrain and hopefully in the ENS against the damage in PD.

### Reporting summary

Further information on research design is available in the Nature Research Reporting Summary linked to this article.

## Supplementary information

Reporting Summary
