# Supplementary material for: Similarities and differences between nigral and enteric dopaminergic neurons unravel distinctive involvement in Parkinson’s disease
Source: NPJ Parkinsons Dis. 2022 Apr 22;8:50. doi: 10.1038/s41531-022-00308-9 (PMC9033791; doi:10.1038/s41531-022-00308-9)
Supplement: Supplementary file 1 — Reporting Summary [file 41531_2022_308_MOESM1_ESM.pdf]

Legend graphic abstract:

- (left) Diagram of ileum and colon, use of original in adaptation; copyright notice: Walsh K.T. & Zemper A.E., “The Enteric Nervous system for Epithelial Researchers: Basic Anatomy, Techniques, and interactions with the epithelium” https://www.sciencedirect.com/science/article/pii/S2352345X19300645?via%3Dihub). Attribution-NonCommercial-NoDerivatives 4.0 International (CC BY-NC-ND 4.0)
- (right) Diagram of specific projections of respectively the VTA neurons to the ventral striatum and of the nigral neurons to the dorsal striatum; use of original in adaptation; credit to: Somayaji M. & Sulzer D., “Investigating pre-clinical dopamine physiology in Parkinson Disease” https://italianacademy.columbia.edu/sites/default/files/papers/IA%20manuscript-MS-final%20(1).pdf. As a co-author Dr. D. Sulzer, agrees to share the diagram now included in the present graphic abstract, which was previously published in the review he co-authored with Dr. Somayaji.
